# Conditional transgenic mice expressing C-terminally truncated human α-synuclein (αSyn119) exhibit reduced striatal dopamine without loss of nigrostriatal pathway dopaminergic neurons

**DOI:** 10.1186/1750-1326-4-34

**Published:** 2009-07-24

**Authors:** João Paulo L Daher, Mingyao Ying, Rebecca Banerjee, Rebecca S McDonald, Myriam Dumas Hahn, Lichuan Yang, M Flint Beal, Bobby Thomas, Valina L Dawson, Ted M Dawson, Darren J Moore

**Affiliations:** 1 NeuroRegeneration and Stem Cell Programs, Institute for Cell Engineering, Johns Hopkins University School of Medicine, Baltimore, USA; 2 Department of Neurology, Johns Hopkins University School of Medicine, Baltimore, USA; 3 Department of Pathology, School of Medicine, Fluminense Federal University, Niterói, Brazil; 4 Department of Neurology and Neuroscience, Weill Medical College of Cornell University, New York, USA; 5 Department of Neuroscience, Johns Hopkins University School of Medicine, Baltimore, USA; 6 Department of Physiology, Johns Hopkins University School of Medicine, Baltimore, USA; 7 Brain Mind Institute, School of Life Sciences, Ecole Polytechnique Fédérale de Lausanne, Lausanne, Switzerland

## Abstract

**Background:**

Missense mutations and multiplications of the *α-synuclein *gene cause autosomal dominant familial Parkinson's disease (PD). α-Synuclein protein is also a major component of Lewy bodies, the hallmark pathological inclusions of PD. Therefore, α-synuclein plays an important role in the pathogenesis of familial and sporadic PD. To model α-synuclein-linked disease *in vivo*, transgenic mouse models have been developed that express wild-type or mutant human α-synuclein from a variety of neuronal-selective heterologous promoter elements. These models exhibit a variety of behavioral and neuropathological features resembling some aspects of PD. However, an important deficiency of these models is the observed lack of robust or progressive nigrostriatal dopaminergic neuronal degeneration that is characteristic of PD.

**Results:**

We have developed conditional α-synuclein transgenic mice that can express A53T, E46K or C-terminally truncated (1–119) human α-synuclein pathological variants from the endogenous murine ROSA26 promoter in a Cre recombinase-dependent manner. Using these mice, we have evaluated the expression of these α-synuclein variants on the integrity and viability of nigral dopaminergic neurons with age. Expression of A53T α-synuclein or truncated αSyn119 selectively in nigrostriatal pathway dopaminergic neurons for up to 12 months fails to precipitate dopaminergic neuronal loss in these mice. However, αSyn119 expression in nigral dopaminergic neurons for up to 12 months causes a marked reduction in the levels of striatal dopamine and its metabolites together with other subtle neurochemical alterations.

**Conclusion:**

We have developed and evaluated novel conditional α-synuclein transgenic mice with transgene expression directed selectively to nigrostriatal dopaminergic neurons as a potential new mouse model of PD. Our data support the pathophysiological relevance of C-terminally truncated α-synuclein species *in vivo*. The expression of αSyn119 in the mouse nigrostriatal dopaminergic pathway may provide a useful model of striatal dopamine depletion and could potentially provide a presymptomatic model of PD perhaps representative of the earliest derangements in dopaminergic neuronal function observed prior to neuronal loss. These conditional α-synuclein transgenic mice provide novel tools for evaluating and dissecting the age-related effects of α-synuclein pathological variants on the function of the nigrostriatal dopaminergic pathway or other specific neuronal populations.

## Background

Parkinson's disease (PD) is the most common neurodegenerative movement disorder characterized by the cardinal symptoms of muscular rigidity, resting tremor and bradykinesia [1,2]. Underlying these motor deficits is the progressive loss of dopaminergic neurons of the substantia nigra pars compacta in addition to several other neuronal populations, the corresponding reduction of striatal dopamine levels, and the appearance of Lewy bodies and Lewy neurites in surviving neurons of the brainstem [1-3]. Lewy bodies are round eosinophilic intracytoplasmic proteinaceous inclusions composed of filamentous material, a major component of which is the protein α-synuclein [4]. Missense mutations (A30P, E46K and A53T) and multiplications of the α-synuclein gene cause rare autosomal dominant familial forms of PD that often manifest disease in some families with an extended clinical and pathological spectrum resembling dementia with Lewy bodies (DLB) [5-9]. Thus, α-synuclein most likely plays a key role in the pathogenesis of familial and sporadic PD in addition to DLB.

It is not clear how mutations in α-synuclein, or increased levels of the wild-type α-synuclein protein due to gene multiplications, precipitate the demise of nigral dopaminergic neurons in familial PD. How α-synuclein contributes to the pathogenesis of the more common sporadic form of PD is also not known. It has been postulated that post-translational modifications of normal α-synuclein, including oxidation, nitration, phosphorylation or C-terminal truncation [10-13], could contribute to α-synuclein dysfunction and neuropathology in sporadic PD. The appearance of some of these modifications correlates well with neuronal loss and/or pathology in sporadic PD brains [11-13]. Furthermore, the exact role of Lewy bodies in dopaminergic neuronal degeneration in PD remains enigmatic. Therefore, clarifying the mechanisms underlying α-synuclein-induced dopaminergic neurodegeneration *in vivo *is critical to understanding the pathogenesis of familial and sporadic PD.

It is now clear from studies of transgenic mouse models that α-synuclein pathogenic mutations induce neuronal dysfunction and degeneration through a toxic gain-of-function mechanism [14-16]. A number of transgenic mice have been created that express wild-type or mutant human α-synuclein in neurons from an array of different heterologous promoter elements. These include the broadly expressing mouse prion protein, mouse Thy-1 and human platelet-derived growth factor-β promoters [17-23], or the catecholaminergic-specific rat tyrosine hydroxylase (TH) promoter [24-27]. A wide range of α-synuclein-related neurological phenotypes have been described in these mouse models that resemble some features of PD, such as behavioral motor deficits, neuronal loss, reduced striatal dopamine levels, and the formation of filamentous and non-filamentous α-synuclein-positive inclusions or aggregates. Many of these phenotypes, however, are not usually observed in the same mouse model. The most severe phenotypes tend to be observed with expression of the A53T variant whereas the wild-type protein is only ever rarely toxic to neurons [15,17,19]. Despite the plethora of α-synuclein mouse models that have been created, only very few of these exhibit a loss of nigral dopaminergic neurons, and in general this neuronal loss is not progressive or robust [26,28]. Therefore, α-synuclein mouse models do not currently exist that reliably recapitulate essential features of PD especially the loss of nigral dopaminergic neurons, the neuropathological hallmark of the disease. Thus, there is a requirement for the development of new α-synuclein transgenic mouse models that can faithfully recapitulate the dysfunction of the nigrostriatal dopaminergic pathway in PD. Such models may arise through improvements in transgenic technology to increase transgene expression levels and dopaminergic neuronal specificity, as well as through the identification of more toxic α-synuclein pathogenic variants. A combination of these approaches could prove to be advantageous in developing faithful α-synuclein mouse models of PD.

Recent studies have highlighted the pathophysiological significance of C-terminally truncated α-synuclein species. Most recently, C-terminal truncation variants, including αSyn119, αSyn122 and αSyn123, have been detected in brain tissue from human PD cases and α-synuclein transgenic mice, and were found to be enriched in Lewy bodies [13,29]. Moreover, *in vitro *studies demonstrate that human α-synuclein lacking the C-terminal 20 or 30 amino acids (αSyn120 or αSyn130) assemble more readily into filaments resembling the α-synuclein filaments observed in Lewy bodies [13,29-31]. Disease-associated mutations in α-synuclein promote the accumulation of these truncated filaments compared to the wild-type protein, and C-terminally truncated species can enhance the aggregation of wild-type α-synuclein at low substoichiometric ratios suggestive of a seeding capacity [13,29,31]. Therefore, C-terminal truncations of α-synuclein could potentially advance disease progression or propagation through promoting the pathological aggregation and accumulation of α-synuclein. At this juncture, however, the role of C-terminally truncated α-synuclein species in the pathogenesis of PD is poorly understood. To further understand the pathophysiological significance of C-terminally truncated α-synuclein species, transgenic mice have recently been developed that express wild-type αSyn120 or A53T mutant αSyn130 truncation variants from the rat TH promoter [25,26]. Collectively, these mice exhibit filamentous and non-filamentous α-synuclein-positive aggregates, deficits in locomotion, reduced striatal dopamine levels, and in the A53T-αSyn130 model a developmental loss of TH-positive nigral neurons and striatal nerve terminals.

Prior to the publication of these α-synuclein truncation models, we set out to develop a collection of transgenic mice capable of producing robust expression of human α-synuclein variants selectively in neurons of the nigrostriatal dopaminergic pathway. Encouraged by the promising neurodegenerative phenotype exhibited by conditional transgenic mice expressing a polyglutamine-expanded huntingtin protein [32], we chose to create similar conditional transgenic mice through gene targeting of a Cre-*lox*P-based transgene cassette at the ROSA26 genomic locus to express E46K or A53T mutant human α-synuclein, or the putative pathogenic C-terminal truncation, αSyn119, in a Cre recombinase-dependent manner. It is worth noting that transgenic mouse models expressing E46K α-synuclein do not currently exist, and that previous α-synuclein truncation models rely upon artificial truncations (αSyn120, αSyn130) [25,26] rather than truncations associated with disease pathology (αSyn119) [13]. Here, we combine conditional transgenic technology with new pathological variants of α-synuclein to evaluate the effects of expressing these α-synuclein variants directly in nigrostriatal pathway dopaminergic neurons.

## Results

### Development of Cre-loxP Conditional Transgenic Mice for Human α-Synuclein Variants

**Figure 1:**
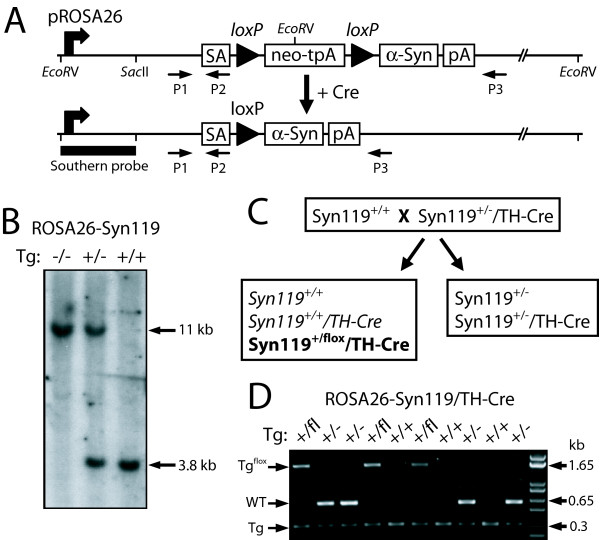
**Generation of Cre-*loxP *Conditional α-Synuclein Transgenic Mice**. (A) Gene targeting strategy for generating conditional ROSA26-αSyn transgenic mice. Human α-synuclein cDNAs were placed downstream of a *loxP*-flanked cassette containing a neomycin resistance gene (neo) and a triple transcriptional termination sequence (tpA). The entire neo-tpA-αSyn conditional transgene cassette is situated adjacent to the endogenous ROSA26 promoter. Following Cre-mediated excision of the neo-tpA cassette, the α-synuclein transgene is expressed from the adjacent ROSA26 promoter. ROSA26-αSyn mice were genotyped using the indicated PCR primers P1, P2 and P3. Primers P1/P3 produce a wild-type band of ~500 bp whereas primers P1/P2 produce a transgenic band of ~250 bp. Following Cre-mediated excision, primers P1/P3 can also amplify a ~1.5 kb band from the recombined transgenic allele. SA, splice acceptor; pA, polyadenylation sequence. (B) Southern blot analysis of *EcoR*V-digested tail genomic DNA derived from either wild-type (-/-), heterozygous (+/-) or homozygous (+/+) ROSA26-αSyn119 mice. Blots were hybridized with a [^32^P]-labeled DNA probe as indicated in (A). The probe detects the wild-type allele at ~11 kb and the transgenic allele at ~3.8 kb owing to an additional *EcoR*V site in the neo gene, as indicated. (C) Breeding strategy to produce floxed ROSA26-αSyn119^+/+^/TH-Cre mice and their non-floxed ROSA26-αSyn^+/+ ^littermates at a frequency of 25% per genotype (*italics*). Notice that homozygous ROSA26-αSyn119^+/+^/TH-Cre mice (*bold*) were often produced with a germ line deletion (flox) of one allele (ROSA26-αSyn119^+/flox^/TH-Cre) at a ratio of 1:1 (+/+ to +/flox) following crossing to TH-Cre mice. (D) PCR genotyping strategy for ROSA26-αSyn119/TH-Cre mice using primers P1, P2 and P3 to distinguish the transgenic allele (Tg, ~250 bp, P1+P2), wild-type allele (WT, ~500 bp, P1+P3), or the transgenic allele following germline deletion of the neo-tpA cassette (Tg^flox^, ~1.5 kb, P1+P3).

To develop new α-synuclein mouse models of PD, our strategy was to create conditional transgenic mice that selectively express pathological human α-synuclein variants in dopaminergic neurons of the nigrostriatal pathway. Therefore, conditional transgenic mice were created that express human A53T or E46K α-synuclein or C-terminally truncated αSyn119 variants from the endogenous murine ROSA26 promoter in a Cre recombinase-dependent manner; hereafter referred to as ROSA26-αSyn mice (Fig. 1A). In these models, a conditional cassette containing an α-synuclein transgene immediately preceded by a *loxP*-flanked transcriptional termination sequence (neo-tpA), has been targeted to the murine ROSA26 locus through homologous recombination [33,34]. Hence, expression of the α-synuclein transgene is driven by the ubiquitous, neuronal-selective murine ROSA26 promoter in discrete neuronal populations following Cre-mediated excision of the neo-tpA cassette (Fig. 1A). Following successful germ line transmission from chimeric mice for each line, the resulting heterozygous progeny (ROSA26-αSyn^+/-^, where + denotes the transgene) were intercrossed and analyzed by Southern blot to confirm correct targeting of the transgene at the ROSA26 locus (Fig. 1B). In general, heterozygous and homozygous ROSA26-αSyn mice are viable and fertile, exhibit normal survival, and manifest no gross behavioral or phenotypic abnormalities.

To induce conditional α-synuclein transgene expression, a two step breeding strategy was developed with either TH-Cre or nestin-Cre transgenic mice to derive homozygous ROSA26-αSyn^+/+ ^mice with or without Cre expression (Fig. 1C). TH-Cre mice display Cre expression and activity restricted to central catecholaminergic neuronal populations within the substantia nigra pars compacta, ventral tegmental area, locus ceruleus, olfactory bulb, hypothalamic nuclei, and superior cervical ganglia [35]. Nestin-Cre mice show widespread expression of Cre activity in neuronal and glial cells throughout the brain [36]. Homozygous ROSA26-αSyn^+/+ ^mice were first crossed with heterozygous ROSA26-αSyn^+/-^/TH-Cre or ROSA26-αSyn^+/-^/nestin-Cre mice in order to eventually produce homozygous ROSA26-αSyn^+/+^/TH-Cre or ROSA26-αSyn^+/+^/nestin-Cre mice and their non-floxed ROSA26-αSyn^+/+ ^littermate controls at an expected frequency of 25% per genotype per litter (Fig. 1C). We notice however that passing the Cre transgene through the germline in this final breeding step results in germline excision of the neo-tpA cassette for one of the transgenic alleles in 100% of the progeny from crosses with nestin-Cre and ~50% of the progeny from crosses with TH-Cre i.e. ROSA26-αSyn^+/flox^/Cre (Fig. 1C, D). This produces homozygous mice with conditional transgene expression from one allele but widespread, unrestricted expression from the opposite allele. This was unexpected since expression of Cre transgenes in these mice is considered to be largely restricted to CNS tissues but they also appear to exhibit some activity in the germline in our mice. Thus, we were restricted to producing cohorts of heterozygous ROSA26-αSyn^+/-^/nestin-Cre mice but could successfully produce homozygous ROSA26-αSyn^+/+^/TH-Cre mice following screening and elimination of mice with germline excision of the neo-tpA cassette (Fig. 1C, D). Collectively, we have created an important resource of conditional transgenic mice capable of expressing A53T, E46K or C-terminally truncated human α-synuclein from the endogenous murine ROSA26 promoter in a Cre recombinase-dependent manner.

### Cre-Dependent Expression of Human A53T α-Synuclein and αSyn119

**Figure 2:**
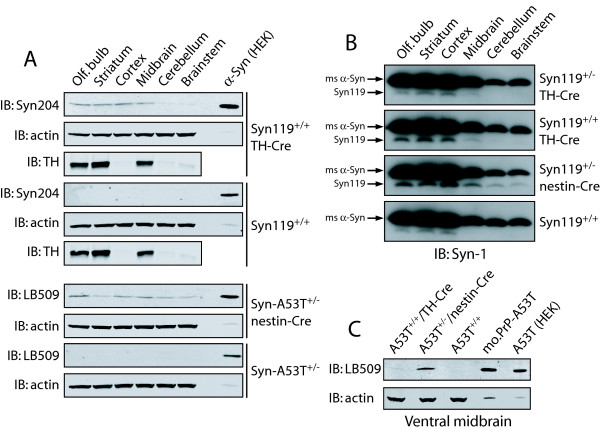
**Cre-Dependent Human α-Synuclein Expression in ROSA26-αSyn Mice**. (A) Cre-dependent expression of α-synuclein variants. Protein extracts (50 μg) derived from distinct brain regions of 10 month-old ROSA26-αSyn119^+/+^/TH-Cre mice and their ROSA26-αSyn119^+/+ ^littermate controls were probed with the human-specific α-synuclein antibody, Syn204, with actin and TH antibodies as controls. HEK-293T cells expressing human αSyn119 were used as a positive control. Equivalent proteins derived from ROSA26-αSyn-A53T^+/-^/nestin-Cre mice and their ROSA26-αSyn-A53T^+/- ^littermates were probed with human-specific α-synuclein antibody, LB509, and actin antibody. HEK-293T cells expressing human αSyn-A53T were used as a positive control. (B) Comparison of human αSyn119 expression with endogenous α-synuclein. Proteins (50 μg) derived from distinct brain regions of 10 month-old ROSA26-αSyn119^+/-^/TH-Cre, ROSA26-αSyn119^+/+^/TH-Cre, ROSA26-αSyn119^+/-^/nestin-Cre and their ROSA26-αSyn119^+/+ ^littermate controls were probed with α-synuclein antibody, Syn-1, to detect endogenous mouse (ms) α-synuclein and human αSyn119 expression. (C) Comparison of human A53T α-synuclein expression in ROSA26-αSyn-A53T mice and mouse prion promoter (mo.PrP) A53T-αSyn transgenic mice. Proteins extracted from ventral midbrain tissue of adult ROSA26-αSyn-A53T mice (50 μg) and mo.PrP-A53T mice (5 μg) were probed with LB509 antibody or actin antibody as a loading control. HEK-293T cells expressing human αSyn-A53T were used as a positive control.

We next sought to verify that α-synuclein transgene expression in these mice can be induced in a Cre-dependent manner. Protein extracts were prepared from brain tissue of 10 month-old floxed ROSA26-αSyn/Cre mice and their non-floxed ROSA26-αSyn littermates followed by Western blot analysis with human-specific α-synuclein antibodies. Human αSyn119 expression is confirmed in brain regions with TH-positive neurons or nerve terminals including the olfactory bulb, striatum, cerebral cortex and ventral midbrain of floxed homozygous ROSA26-αSyn119^+/+^/TH-Cre mice that is completely absent from non-floxed littermate mice (Fig. 2A). Similarly, the expression of A53T human α-synuclein is confirmed in brain regions with nestin-positive neurons from floxed heterozygous ROSA26-αSyn-A53T^+/-^/nestin-Cre mice that is absent from the brains of their non-floxed littermate mice (Fig. 2A). Thus, both A53T α-synuclein and αSyn119 transgenes are expressed in a Cre-dependent manner in these mice. The E46K α-synuclein transgenic mice were not characterized further in this study.

To compare the expression level of human αSyn119 in the ROSA26-αSyn mice with endogenous α-synuclein, proteins extracted from various brain regions of 10 month-old ROSA26-αSyn119/Cre mice and their non-floxed littermates were analyzed by Western blotting with the α-synuclein antibody, Syn-1. αSyn119 is expressed at less than 10% of the levels of endogenous α-synuclein in these brain regions (Fig. 2B). This is perhaps not surprising since αSyn119 is expressed selectively in most TH-positive catecholaminergic neurons in these brain regions whereas endogenous α-synuclein is known to be expressed broadly within many neuronal populations throughout the brain [37]. It is possible to observe a doubling of α-Syn119 expression between heterozygous and homozygous floxed ROSA26-αSyn119/TH-Cre mice as expected (Fig. 2B). We could also confirm a broader distribution and higher levels of αSyn119 in ROSA26-αSyn119^+/-^/nestin-Cre mice compared to ROSA26-αSyn119^+/+^/TH-Cre mice reflecting the widespread distribution and greater number of nestin-positive neurons compared to the more restricted TH-positive catecholaminergic neuronal population in each brain region (Fig. 2B). A small proportion of truncated endogenous α-synuclein could also be detected in forebrain regions from control ROSA26-αSyn119^+/+ ^mice that express the highest levels of endogenous α-synuclein (Fig. 2B), as noted previously [13]. It was not possible to directly compare the expression level of human A53T α-synuclein with endogenous α-synuclein in the ROSA26-αSyn-A53T/Cre mice since these proteins co-migrate and human α-synuclein expression does not appreciably influence the total level of α-synuclein detectable using the Syn-1 antibody (data not shown).

Finally, we compared the expression level of A53T α-synuclein in our ROSA26-αSyn mice to a well-characterized mouse prion promoter (mo.PrP)-based A53T human α-synuclein neurodegenerative mouse model [19,38]. Despite the lack of obvious substantia nigra pathology in this mo.PrP-A53T mouse model, it is possible to detect more prominent α-synuclein expression in the ventral midbrain by Western blotting with the human-specific LB509 antibody compared to the ROSA26-αSyn-A53T mice with transgene expression directed to either TH-positive or nestin-positive neurons (Fig. 2C). This finding most likely relates to the relatively small contribution of TH-positive neurons to the total number of neurons within the ventral midbrain region which is highlighted by the observation that *heterozygous *ROSA26-αSyn-A53T^+/-^/nestin-Cre mice produced far greater transgene expression than *homozygous *ROSA26-αSyn-A53T^+/+^/TH-Cre mice (Fig. 2C). Greater α-synuclein expression observed in the mo.PrP-A53T transgenic mice compared to the ROSA26-αSyn-A53T^+/-^/nestin-Cre mice most likely relates to differences in the strength of the ROSA26 and mo.PrP promoters and/or transgene copy number, both of which are considered to be higher in the mo.PrP-A53T mice [19]. Due to the low level of transgene expression in the ROSA26-αSyn/Cre mice it was not possible to reliably demonstrate the selective neuronal distribution pattern of human α-synuclein above background by immunohistochemical methods with currently available human-specific α-synuclein antibodies or the Syn-1 antibody (data not shown). It was also not possible to observe the presence of α-synuclein pathological inclusions or aggregates in the substantia nigra of these mice up to 10 months of age (data not shown). Collectively, this data demonstrates that the ROSA26-αSyn mice express human αSyn119 or human A53T α-synuclein in a Cre-dependent manner but at lower levels than those of endogenous, murine α-synuclein or A53T α-synuclein expressed in mo.PrP transgenic mice.

### Lack of Nigral Dopaminergic Neuronal Degeneration in α-Synuclein Transgenic Mice

**Figure 3:**
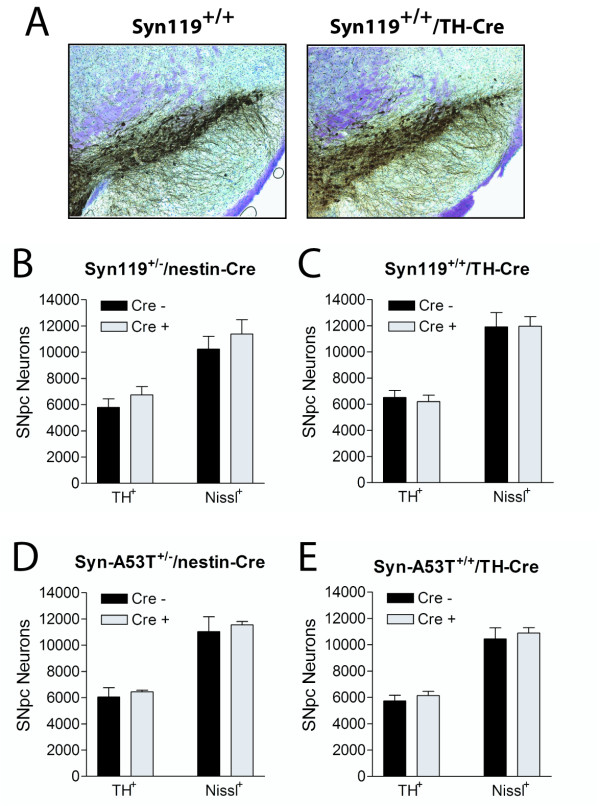
**Lack of Nigral Dopaminergic Neuronal Degeneration in ROSA26-αSyn Mice**. Stereological analysis of TH-positive and Nissl-positive neurons in the substantia nigra pars compacta of male ROSA26-αSyn mice with or without Cre expression. (A) Example of immunohistochemical staining for TH with Nissl counterstain on coronal midbrain sections highlighting the substantia nigra region from 12 month-old ROSA26-αSyn119^+/+^/TH-Cre mice and their ROSA26-αSyn119^+/+ ^littermate controls. (B-E) Stereological analysis revealing a normal number of TH-positive and Nissl-positive neurons in the substantia nigra pars compacta (SNpc) of 10–12 month-old ROSA26-αSyn119^+/-^/nestin-Cre (B), ROSA26-αSyn119^+/+^/TH-Cre (C), ROSA26-αSyn-A53T^+/-^/nestin-Cre (D) and ROSA26-αSyn-A53T^+/+^/TH-Cre (E) mice compared to their age-matched non-floxed ROSA26-αSyn littermates (Cre -). Data are expressed as the mean ± SEM (*n *= 4–6 mice per genotype). There are no significant differences (p > 0.05) between genotypes following statistical analysis by unpaired, two-tailed Student's *t *test.

To determine whether the expression of human αSyn119 or A53T α-synuclein in the ROSA26-αSyn/Cre mice could contribute to the degeneration of nigrostriatal dopaminergic neurons with age, we generated suitably-sized cohorts of aged floxed and non-floxed mice. In particular, we generated αSyn119 or A53T α-synuclein mice crossed with TH-Cre (ROSA26-αSyn^+/+^/TH-Cre) or nestin-Cre (ROSA26-αSyn^+/-^/nestin-Cre) transgenic mice and their non-floxed age-matched littermates (ROSA26-αSyn^+/- ^or ROSA26-αSyn^+/+ ^mice). It is anticipated that TH-Cre and nestin-Cre mice would direct transgene expression in the vast majority of nigral dopaminergic neurons in the ROSA26-αSyn/Cre mice [35,36]. Mice were aged to 10–12 months and the numbers of TH-positive and Nissl-positive neurons in the substantia nigra pars compacta were counted using unbiased stereological methods (Fig. 3A). At this age, we fail to observe a significant loss of nigral dopaminergic neurons or Nissl-positive neurons due to human αSyn119 or A53T α-synuclein expression (Fig. 3A–E). In general, we do not observe alterations in the survival of the ROSA26-αSyn119/Cre mice up to 18 months of age. It is not possible to conclude whether or not the αSyn119 protein has pathophysiological properties since comparable mice expressing pathogenic A53T α-synuclein at identical levels in the same neurons also failed to influence dopaminergic neuronal viability in these mice.

### Reduced Striatal Dopamine Induced by αSyn119 Expression in Catecholaminergic Neurons

**Figure 4:**
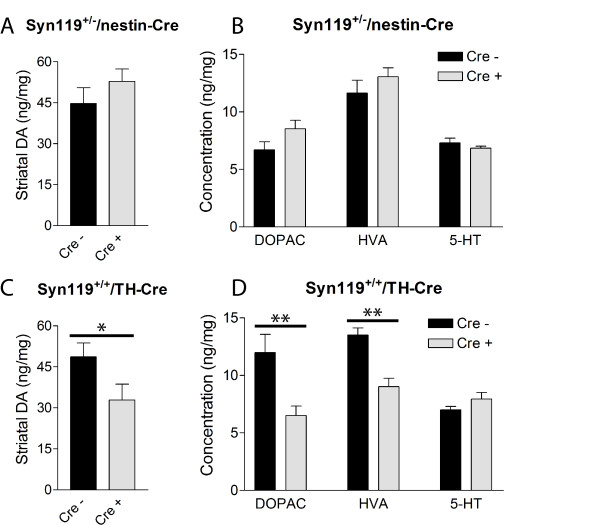
**αSyn119 Expression in Catecholaminergic Neurons Leads to a Reduction of Striatal Dopamine and its Metabolites**. Analysis of striatal biogenic amines in ROSA26-αSyn119 mice by HPLC with electrochemical detection. The level of dopamine (A, C), or the dopamine metabolites, DOPAC and HVA, and 5-HT (B, D) were measured by HPLC in striatal tissue from (A, B) 12–13 month-old female ROSA26-αSyn119^+/-^/nestin-Cre mice (Cre +) and their ROSA26-αSyn119^+/- ^littermate controls (Cre -) and from (C, D) 10–11 month-old female ROSA26-αSyn119^+/+^/TH-Cre mice (Cre +) and their ROSA26-αSyn119^+/+ ^littermate controls (Cre -). The concentration of each biogenic amine is expressed as ng per mg of protein, and data represent the mean ± SEM (*n *= 7–9 mice for nestin-Cre and *n *= 5–6 mice for TH-Cre). Significant differences between Cre- and Cre + genotypes were determined following statistical analysis by unpaired, two-tailed Student's *t *test (*p < 0.05, **p < 0.01).

To determine whether the expression of human αSyn119 in nigral dopaminergic neurons leads to nigrostriatal neuronal dysfunction, we monitored the levels of striatal dopamine and its metabolites in aged ROSA26-αSyn119/Cre mice and their non-floxed littermate control mice by HPLC. No differences in the levels of striatal dopamine or its metabolites, 3,4-dihydroxyphenylacetic acid (DOPAC) and homovanillic acid (HVA), are observed in heterozygous ROSA26-αSyn119^+/-^/nestin-Cre mice compared to their non-floxed littermates at 12–13 months of age (Fig. 4A, B). However, in homozygous ROSA26-αSyn119^+/+^/TH-Cre mice with an approximate doubling of αSyn119 expression in TH-positive dopaminergic neurons, we observe a significant marked reduction in the levels of striatal dopamine (~32%), DOPAC (~46%) and HVA (~33%) compared to their non-floxed ROSA26-αSyn119^+/+ ^littermates at 10–11 months of age (Fig. 4C, D). This effect on the nigrostriatal dopaminergic system is specific since the levels of striatal 5-hydroxytryptamine (5-HT) in these ROSA26-αSyn119^+/+^/TH-Cre mice are normal (Fig. 4D). No further alterations in dopamine levels in other regions of the brain are observed in these mice, including the olfactory bulb, cerebral cortex and prefrontal cortex (data not shown). Furthermore, striatal dopamine turnover in the ROSA26-αSyn119/Cre mice is also normal (data not shown). Taken together, this data demonstrates that the expression of human αSyn119 in TH-positive catecholaminergic neurons leads to a reduction in striatal dopamine content in the absence of nigral dopaminergic neuronal loss. This effect would appear to be dependent on transgene dosage since the reduced expression of αSyn119 in heterozygous ROSA26-αSyn119^+/-^/nestin-Cre mice fails to induce deficits in striatal dopamine levels. Alternatively, this effect could potentially relate to mosaic expression of Cre recombinase in nigral dopaminergic neurons of TH-Cre and nestin-Cre transgenic mice. Our data suggest that human αSyn119 may be of pathophysiological relevance *in vivo*.

### Neurochemical Alterations in αSyn119 Transgenic Mice

**Figure 5:**
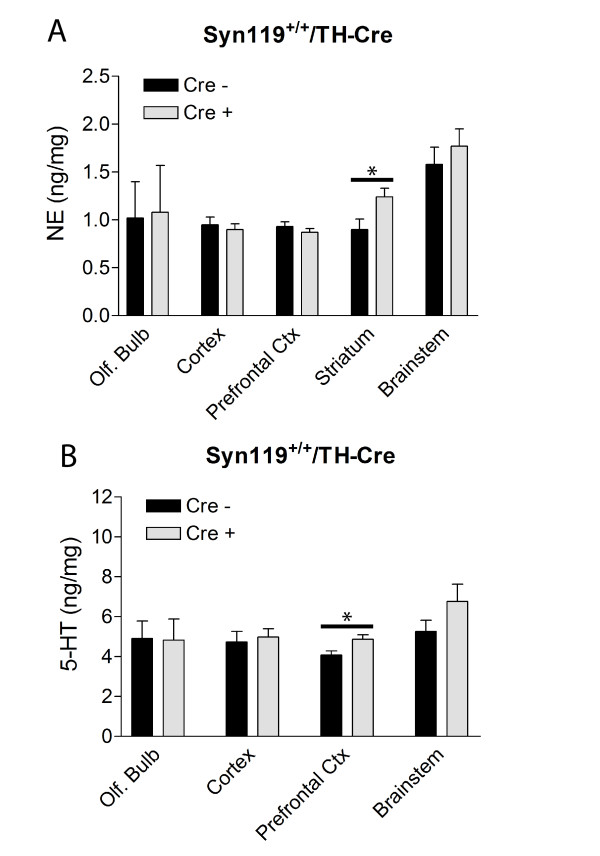
**Neurochemical Alterations Caused by αSyn119 Expression in Catecholaminergic Neurons**. Analysis of biogenic amines in discrete brain regions of ROSA26-αSyn119 mice by HPLC with electrochemical detection. The level of NE (A) and 5-HT (B) were measured by HPLC in olfactory bulb, cerebral cortex, prefrontal cortex, striatum and brainstem tissues of 10–11 month-old female ROSA26-αSyn119^+/+^/TH-Cre mice (Cre +) and their ROSA26-αSyn119^+/+ ^littermate controls (Cre -). The concentration of NE and 5-HT is expressed as ng per mg of tissue, and data represent the mean ± SEM (*n *= 5–7 mice per genotype). Significant differences between Cre - and Cre + genotypes were determined following statistical analysis by unpaired, two-tailed Student's *t *test (*p < 0.05).

To further evaluate the potential pathophysiological effects of αSyn119 expression in the brain, the levels of the neurotransmitters norepinephrine (NE) and 5-HT were monitored in the olfactory bulb, cerebral cortex, prefrontal cortex, striatum and brainstem. A significant increase in the level of NE in the striatum (Fig. 5A) and 5-HT in the prefrontal cortex (Fig. 5B) is observed in homozygous ROSA26-αSyn119^+/+^/TH-Cre mice compared to their non-floxed littermates at 10–11 months of age. NE and 5-HT levels in these brain regions are normal in heterozygous ROSA26-αSyn119^+/-^/nestin-Cre mice at 12–13 months of age suggesting that the alterations in neurotransmitter levels are specific to the homozygous ROSA26-αSyn119^+/+^/TH-Cre mice (data not shown). Thus, αSyn119 expression in TH-positive catecholaminergic neurons induces additional subtle neurochemical alterations *in vivo*.

## Discussion

Here, we describe the development and evaluation of conditional transgenic mice expressing human α-synuclein pathological variants in dopaminergic neurons of the nigrostriatal pathway from the endogenous ROSA26 promoter. Our investigations have focused on the potential contribution of two pathologic variants, A53T mutant α-synuclein and C-terminally truncated αSyn119, to the normal integrity and function of nigral dopaminergic neurons with age. Our studies demonstrate that A53T α-synuclein and αSyn119 are expressed in a Cre recombinase-dependent manner in these ROSA26-αSyn mice. We show that neither A53T α-synuclein nor αSyn119 expression in mice aged for up to 12 months is sufficient to induce the degeneration of nigral dopaminergic neurons. However, we demonstrate that the expression of αSyn119 in nigral dopaminergic neurons for up to 12 months in this mouse model causes a marked reduction in the levels of striatal dopamine and its metabolites in addition to other subtle neurochemical alterations. Thus, at ages up to 12 months we observe nigrostriatal dopaminergic pathway dysfunction in the absence of frank neuronal loss. Further deficits in nigrostriatal dopaminergic function may be revealed in these mice with advanced age. The ROSA26-αSyn mice therefore represent a useful model of striatal dopamine depletion and could provide a potential presymptomatic model of PD perhaps representative of the earliest derangements in dopaminergic neuronal function observed prior to neuronal loss.

Our data suggest that the C-terminally truncated α-synuclein variant, αSyn119, is of pathophysiological relevance *in vivo*. The expression of αSyn119 in nigral TH-positive dopaminergic neurons precipitates striatal dopamine loss without compromising dopaminergic neuronal viability. αSyn119, together with αSyn122 and αSyn123, are normally present in mammalian brain at low levels but are enriched as soluble and aggregated species in Lewy body-positive PD and DLB brains and in transgenic mouse brains expressing human A53T α-synuclein [13,29]. Furthermore, disease-associated mutations in α-synuclein promote the accumulation of αSyn119 and other truncation species compared to the wild-type protein in neural cell lines and α-synuclein transgenic mice [13,29]. Further supporting the pathogenicity of truncated αSyn119 and related species, *in vitro *studies demonstrate that C-terminally truncated α-synuclein variants fibrillize and aggregate more readily than the full-length protein and can seed the aggregation of full-length α-synuclein [13,29-31]. The mechanism through which α-synuclein C-terminal truncations arise is not known. α-Synuclein can be proteolytically processed at its C-terminus via a number of proteases *in vitro*, including cathepsin D and calpain I, but as yet none of these proteases have been shown to specifically generate the αSyn119 variant [39-42]. However, αSyn119 expression has been demonstrated *in vivo *and this variant accumulates in diseased brain tissue [13,29]. The normal and pathophysiological mechanism responsible for the generation of αSyn119 and similar truncation variants *in vivo *remains to be clarified.

Our data in the ROSA26-αSyn mice provides the first *in vivo *demonstration of a pathophysiological effect of the disease-associated αSyn119 species on the nigrostriatal dopaminergic pathway. It is not yet clear how the expression of αSyn119 in dopaminergic neurons produces deficits in striatal dopamine levels in the absence of frank neuronal loss. αSyn119 could potentially induce the dysfunction or degeneration of striatal dopaminergic nerve terminals without compromising the integrity of the neuronal soma. At the cellular level, αSyn119 could impair pre-synaptic dopaminergic nerve terminals through the accumulation of submicroscopic toxic oligomers or aggregates [13,29], or could potentially sensitize nerve terminals to secondary insults such as oxidative and nitrosative stress that accumulate with age [43]. Future analyses of these mice will aim to clarify the mechanism through which αSyn119 could potentially act at nigrostriatal dopaminergic nerve terminals. The effects of αSyn119 expression *in vivo *are reminiscent to those of αSyn120 expressed in transgenic mice from the rat TH promoter on an α-synuclein null background [25]. αSyn120 transgenic mice exhibit a reduction in striatal dopamine and HVA levels that are first evident between 1–3 months but do not worsen up to 12 months of age [25]. Accompanying these alterations are late-onset motoric deficits and the formation of pathological α-synuclein inclusions. A caveat of this mouse model is that the αSyn120 truncated species has not yet been observed *in vivo *in brain tissue and may therefore represent a non-physiological truncated α-synuclein species [13]. Furthermore, the αSyn120 mice were generated on an α-synuclein null background which complicates the interpretation of their phenotype. Nevertheless, the ROSA26-αSyn119 mice and the αSyn120 transgenic mice suggest that C-terminal truncation of α-synuclein could represent a pathophysiological process during the development and/or propagation of PD that potentially contributes to the dysfunction of the nigrostriatal dopaminergic pathway.

The ROSA26-αSyn mice offer an important resource for modeling various aspects of PD. These mice allow the expression of disease-associated α-synuclein variants selectively in nigrostriatal pathway dopaminergic neurons in order to evaluate their potentially pathogenic affects with age. Transgene expression in these mice is driven by the well-characterized neuronal-specific, endogenous murine ROSA26 promoter in a Cre-dependent manner [33,34,44]. This system permits the expression of α-synuclein variants in virtually any neuronal population in the brain depending upon the availability of suitable Cre driver lines. Conceivably, these ROSA26-αSyn mice could also be used to model other α-synucleinopathies, such as DLB, by directing transgene expression to neurons of the forebrain. A particularly attractive feature of these ROSA26-αSyn mice is that one can directly compare the effects of different pathological variants of the α-synuclein protein since variants are expressed at equivalent levels from the same stable promoter at identical copy number in the same neurons. This provides a clear advance over conventional transgenesis where individual founder lines can often produce marked differences in transgene expression levels and expression patterns due to the effects of genomic integration site, epigenetic modifications and transgene copy number. Of note, there are no known phenotypes associated with disruption of the ROSA26 genomic locus [33,44]. However, an obvious caveat of the ROSA26-αSyn mice is reduced transgene expression since only one to two transgene copies can be accommodated at the ROSA26 locus. Using the ROSA26-αSyn mice it is possible to co-express two pathological α-synuclein variants from opposite alleles at equivalent levels in the same neurons to investigate how they may interact to precipitate pathology or compromise neuronal viability. This feature allows one to examine, for example, whether αSyn119 expression can seed and accelerate the aggregation of the full-length α-synuclein variants, A53T or E46K, in dopaminergic neurons *in vivo*. Another useful feature of the ROSA26-αSyn mice is that they are ideally suited for investigating potential gene-environment interactions with dopamine neuron-specific toxins such as MPTP [45] since α-synuclein expression can be directed selectively to nigrostriatal pathway dopaminergic neurons using the TH-Cre driver line. In this scenario, one can directly compare the potential differential interaction of dopaminergic toxins with various pathogenic α-synuclein variants. Importantly, we have made these ROSA26-αSyn mice available as a unique resource to the PD research community by distribution through The Jackson Laboratory. The ROSA26-αSyn mice provide a useful and unique tool for evaluating the age-related effects of expressing α-synuclein pathogenic variants in neurons *in vivo *that will hopefully provide important insight into the molecular mechanisms underlying the pathogenesis of PD and DLB.

## Materials and methods

### Generation and Breeding of Conditional α-Synuclein Transgenic Mice

Human α-synuclein variants (E46K, A53T or 1–119) were targeted to the ROSA26 genomic locus in *129/SvJ *embryonic stem (ES) cells as described [34]. Briefly, α-synuclein cDNAs were subcloned into *Xho*I and *Not*I sites of acceptor plasmid pBigT thereby placing the transgene downstream of a *loxP*-flanked neo-tpA transcriptional termination cassette [34]. The entire neo-tpA-α-synuclein conditional transgene was excised and inserted into targeting plasmid pROSA26PA via *Pac*I and *Asc*I sites [34]. The final pROSA26PA-αSyn construct was linearized with *Kpn*I, gel purified with GELase reagent (Epicenter, Madison, WI) and used for electroporation of mouse *129/SvJ *ES cells. Following G418 selection, at least six correctly targeted ES cell clones were identified by PCR and Southern blot analysis as described [33,34], and two clones were microinjected into *C57BL/6J *mouse blastocysts to derive chimeric mice. Four to six chimeras were bred with *C57BL/6J *mice for each targeting construct with at least one chimeric mouse producing germline transmission of the correctly targeted allele to F1 progeny. ES cell selection, blastocyst microinjection and chimera breeding was conducted by the University of Cincinnati Gene-Targeted Mouse Service. F1 heterozygous mice were routinely identified by PCR of tail genomic DNA with primers: P1, 5'-aaagtcgctctgagttgttat-3'; P2, 5'-gcgaagagtttgtcctcaacc-3'; P3, 5'-ggagcgggagaaatggatatg-3'; producing a ~500 bp product from the WT allele and a ~250 bp product from the transgenic allele [33,34]. Following intercrossing of F1 heterozygous mice, mice were further analyzed by Southern blot to verify correct targeting of the transgene at the ROSA26 locus.

ROSA26-αSyn mice were maintained as *129/SvJ *and *C57BL/6J *hybrids through backcrossing to *C57BL/6J *mice for 1–2 generations and then intercrossing of heterozygous progeny. TH-Cre [35] and nestin-Cre [36] transgenic mice were maintained on a *C57BL/6J *background. Nestin-Cre transgenic mice (stock number 003771) were obtained from The Jackson Laboratory (Bar Harbor, ME). To generate cohorts of mice for aging, heterozygous ROSA26-αSyn^+/- ^mice were crossed with each hemizygous Cre line, and the F1 ROSA26-αSyn^+/-^/Cre progeny were further crossed with homozygous ROSA26-αSyn^+/+ ^mice to produce F2 mice. The F2 mice produced from these crosses on a mixed *129/SvJ *and *C57BL/6J *hybrid background were used throughout this study. Age-matched ROSA26-αSyn^+/- or, +/+ ^littermates without Cre were used as controls. Cre transgenes were genotyped by an established PCR protocol with the primers 5'-AAATGTTGCTGGATAGTTTTTACTGC-3' and 5'-GGAAGGTGTCCAATTTACTGACCGTA-3' to produce a 300 bp transgenic fragment [35]. Adult hemizygous PrP-A53T-αSyn transgenic mice [19] were kindly provided by Dr. Michael K. Lee (Johns Hopkins University). All mice were housed and treated in strict accordance with the National Institutes of Health *Guide for the Care and Use of Laboratory Animals*. Mice were maintained in a pathogen-free facility and exposed to a 12 h light/dark cycle with food and water provided *ad libitum*. The ROSA26-αSyn mice are available from The Jackson Laboratory (Bar Harbor, ME; ); JAX stock number, 008883 (ROAS26-Syn-A53T), 008886 (ROSA26-Syn-E46K) and 008889 (ROSA26-Syn119).

### Western blot analysis

Brain regions, including the olfactory bulb, cerebral cortex, striatum, ventral midbrain, brainstem and cerebellum, from 10 month-old mice were dissected from fresh brains, homogenized in ice-cold RIPA buffer (50 mM Tris-HCl, pH 7.4, 150 mM NaCl, 5 mM EDTA, 1% Triton X-100, 0.1% SDS, 1× Complete Mini protease inhibitor cocktail [Roche]), and then centrifuged at 20,000 × *g *for 15 min at 4°C. The RIPA-soluble supernatant fraction was collected, quantified using BCA reagent (Pierce, Rockford, IL) with BSA standards, and stored at -80°C. Proteins (100 μg) were resolved by electrophoresis on 15% SDS-PAGE gels and transferred to nitrocellulose membranes (0.2 μm; Biorad). To detect α-synuclein expression, blots were probed with human-specific mouse monoclonal antibodies, Syn204 (Cell Signaling Technology) and LB509 (Zymed), or with the mouse monoclonal Syn-1 antibody (BD Bioscience). Antibodies to tyrosine hydroxylase (Novus Biologicals) and actin (Sigma) were used to control for regional dissection and protein loading, respectively. Lysates derived from HEK-293T cells transiently expressing untagged human α-synuclein (A53T or Syn119; pcDNA3.1 expression vector [Invitrogen] [19]) served as positive controls for α-synuclein antibodies. To compare A53T α-synuclein expression in ventral midbrain tissue from adult mo.PrP-A53T-αSyn mice [19] and ROSA26-αSyn-A53T/Cre mice, proteins were prepared as described above and analyzed by Western blotting with LB509 antibody.

### Immunohistochemistry and Stereological Cell Counting

Perfusion-fixed coronal midbrain sections (40-μm) were cut on a sliding microtome (Microm, Kalamazoo, MI) and collected free-floating in PBS. Sections were permeabilized and blocked in PBS containing 0.4% Triton X-100 and 4% normal goat serum at room temperature for 30 min. Sections then were incubated in rabbit polyclonal anti-TH antibody (1:1000; Novus Biologicals, Littleton, CO) in PBS containing 0.2% Triton X-100 and 2% normal goat serum overnight at 4°C and washed with PBS containing 0.2% Triton X-100 and 1% normal goat serum. Next, sections were incubated for 1 hr in biotinylated goat anti-rabbit antibody (1:1000; Jackson ImmunoResearch, West Grove, PA) in PBS with 0.2% Triton X-100 and 1.5% goat serum, washed, and visualized by incubation in biotin-streptavidin-HRP complex (ABC; Vector Laboratories, Burlingame, CA), followed by incubation with 3,3'-diaminobenzidine per the manufacturer's instructions (Sigma). Sections were mounted on glass slides and allowed to air dry overnight before being counterstained with Nissl, dehydrated with ethanol, and cover-slipped for visualization. To evaluate dopaminergic neuronal loss, unbiased stereological methodology was employed as described previously [46,47] to count TH-positive and Nissl-positive neurons in the left and right pars compacta region of every fourth section throughout the entire midbrain region. This method was carried out by using a computer-assisted image analysis system, consisting of an Axiophot 2 photomicroscope (Carl Zeiss Vision, Hallbergmoos, Germany) equipped with a computer-controlled motorized stage (Ludl Electronics, Hawthorne, NY), a Hitachi HV C20 video camera, and interfaced with a Stereo Investigator system (MicroBrightField, Williston, VT, USA) with optical fractionator probe.

### Measurement of Biogenic Amines by HPLC

HPLC with electrochemical detection was used to measure the concentration of the biogenic amines, dopamine, 3,4-dihydroxyphenylacetic acid (DOPAC), homovanillic acid (HVA), 5-hydroxytryptamine (5-HT), 5-hydroxyindoleacetic acid (5-HIAA) and norepinephrine (NE) in discrete brain regions, as previously described [48]. Female ROSA26-αSyn/Cre mice and age-matched ROSA26-αSyn littermate controls were sacrificed by decapitation, brains were quickly removed and frozen on dry ice, and stored at -80°C. Striata, prefrontal cortex, cerebral cortex, olfactory bulb and brainstem were dissected from frozen tissue and samples were weighed and sonicated in 0.2 ml of 0.1 M perchloric acid (at 100 μl/mg tissue) containing 0.01% EDTA and 25 μg/ml 3,4-dihydroxybenzylamine (DHBA, Sigma) as an internal standard. Following centrifugation at 15,000 × *g *for 10 min at 4°C, 20 μl of supernatant was injected onto a C-18 80 × 4.6 mm column (ESA, Inc Chelmsford, MA). The mobile phase consisted of 0.1 M LiH_2_PO_4_, 0.85 mM 1-octanesulfonic acid and 10% (v/v) methanol. The flow rate was kept at 1 ml/min. Biogenic amines and their metabolites were detected by a 2-channel Coulochem II electrochemical detector (ESA, Inc. Chelmsford, MA) with the working electrode kept at 0.7 V. Data were collected and processed using external standards for respective amines on a EZChrome Elite Client Workstation (ESA, Inc. Chelmsford, MA). Concentrations of biogenic amines are expressed as ng per mg protein. The protein concentrations of tissue homogenates were measured using BCA reagent (Pierce, Rockford, IL).

### Statistical Analysis

Throughout the experiments investigators were blinded to genotype of the mice. Data represent mean ± S.E.M. from groups of animals for each genotype. Statistical analysis for stereology and HPLC assessments between genotypes were assessed by unpaired, two-tailed Students *t *test. Differences were considered significant when *p *< 0.05. All statistical analyses were performed using GraphPad InStat-version 3 and Prism software (San Diego, CA).

## Abbreviations

DLB: dementia with Lewy bodies; DOPAC: 3,4-dihydroxyphenylacetic acid; 5-HT: 5-hydroxytryptamine; HVA: homovanillic acid; NE: norepinephrine; neo: neomycin; PD: Parkinson's disease; TH: tyrosine hydroxylase; tpA: transcriptional termination sequence.

## Competing interests

The authors declare that they have no competing interests.

## Authors' contributions

TMD and DJM designed the research, JPD, MY, RB, RSM, LY and BT conducted the experiments, TMD and DJM contributed new research tools, MDH, BT, MFB, VLD, TMD and DJM supervised the experiments and provided reagent and infrastructure support, JPD, MY, BT, TMD and DJM analyzed the data, DJM prepared the manuscript, BT, MFB, VLD and TMD critically reviewed the manuscript. All authors read and approved the final manuscript.
